# Treatment of Lachrymal Obstruction

**Published:** 1935

**Authors:** Gordon R. Scarff

**Affiliations:** Assistant Surgeon, Ear, Nose and Throat Department, Bristol General Hospital


### TREATMENT OF LACHRYMAL OBSTRUCTION.

BY

GORDON R. SCARFF, M.B., CH.B., F.R.C.S.,

*Assistant Surgeon, Ear, Nose and Throat Department,*
*Bristol General Hospital.*

---

IN affections of the lachrymal sac the rhinologist may be called in to ascertain whether there is any predisposing cause in the nose and to treat this if found, or to treat the condition of the sac when this involves manipulations in an area with which he is familiar. There is close relationship between the lachrymal sac and the nasal cavity, in addition to direct communication through the tear duct, and although ascending infection from the nose may to some extent be prevented by folds of mucous membrane (so-called valves), yet, on account of this close relationship, affections of the sac are not infrequently found to be of nasal origin.

TYPES OF CASES REFERRED TO THE RHINOLOGIST.

1. Taking first the reflex epiphora to which Mr. Iles referred, this, in my experience, is usually an allergic condition, and its treatment would require a discussion in itself.

2. Lachrymal obstruction due to causes within the nose. These usually occur as malignant growths, expecially of the antrum or ethmoid, and treatment is that of the causal condition.

3. Lachrymal obstruction due to stenosis of the lachrymal duct.

In nearly half the cases which have been referred to me there has been marked nasal obstruction on the affected side, due to deflection of the septum, while in two further cases there has been suppuration in the nasal accessory sinuses. Treatment of the nasal condition alone was tried in three cases, but failed to relieve the epiphora, and further treatment of the obstruction was necessary.

Until recently at the Bristol Eye Hospital, as at many others, excision of the sac has been regarded as the most satisfactory treatment in cases where conservative measures have failed to bring about a cure, although it has been felt that this is not the ideal surgical treatment, as the results are not invariably satisfactory, and it fails to restore the natural function. With the latter end in view we decided to employ measures aimed at re-establishing the flow of tears into the nose, which must be done by effecting a direct communication between the sac and the nasal fossa. At first sight this procedure would seem easy enough, as there is only the thin plate of the lachrymal bone separating a large portion of the lachrymal sac from the nasal mucosa, which is easily broken through and a fistula made; but as a hole made to remain open always tries its best to shut, whatever operative procedure is adopted a large hole must be made, and the minimum of damage must be done to surrounding tissues in order to prevent excessive scar tissue formation.

This problem is by no means a new one. Surgical treatment of dacryocystitis and epiphora is recorded as early as the time of Celsus in the first century A.D., who recommended complete removal of the sac, followed by the plunging of a red-hot iron through the lachrymal fossa in order to make communication with the nose. This method was later modified by the drilling of one or more holes into the nose, and was kept up until the seventh century, when the second part of the operation was given up. Little more is recorded of surgical procedures until the eighteenth century, when Anel first probed the duct and syringed out the lachrymal sac. Later, extirpation of the lachrymal sac was the treatment of choice, although attempts were made to fistulise the sac into the nose by means of glass and silver cannulæ passed through a slit canaliculus. In 1904 Toti introduced his operation of external dacryocystorhinostomy, operating through an incision in the lower lid. A few years later West and Polyak evolved the operation of intra-nasal dacryocystorhinostomy.

Since that time each of the three methods has had its supporters, a plain indication that no one of them is universally successful, and that the perfect treatment has yet to be found. Indeed, West himself, in dealing with small fibrous sacs, has returned to the treatment of Celsus, though in less barbarous a manner, that is, by his intranasal method, removing the entire sac and fistulising the canaliculi directly into the nose.

Mr. Iles has already told you of the indications for removal of the sac, to which I should like to add the conclusions drawn from my short experience of the other methods.

### West's Operation.

This consists in turning down a flap of nasal mucosa in front of the middle turbinate, removing the bone over the sac with a chisel, and excising the inner wall of the sac.

*Advantages.*—No external scar, no possibility of infecting perilachrymal tissues.

*Disadvantages.* — Narrow access, difficulty of breaking through the thick frontal process of the superior maxilla, ethmoidal cells overlying the area of the sac may render access even more difficult. Submucous resection of the septum is usually required as a preliminary.

### TOTI OPERATION.

In this operation access is gained to the sac through an external incision in the lower lid in the line of the anterior lachrymal crest. The sac is retracted outwards and its bony bed removed by chisel with a generous margin antero-inferiorly. The internal wall of the sac is removed and a corresponding area of nasal mucosa also taken away. The original operation is now modified in that the incision is not carried through the internal palpebral ligament, and also the anterior end of the middle turbinate is amputated if it encroaches at all on the field of operation.

*Advantages.*—Every step can be clearly seen and the outline of the sac clearly defined. The space in which one is working, although small, is not at a great depth from the surface, and one can remove with precision the necessary portions of the lachrymal crest and the thick frontal process of the maxilla.

*Disadvantages.*—The operation in the majority of cases leaves an almost invisible scar, but where there is an empyema of the sac there is a possibility of infection of the surrounding tissues, which may cause the breaking down of the external incision with slight broadening of the scar, as well as contraction and possible closure of the opening into the nose.

*Difficulties common to both operations.*—Difficulty may arise:

(1) Where the sac is small and fibrotic, or

(2) when there is already a considerable amount of fibrous tissue intervening between the sac and the nasal wall as a result of old suppuration.

*Contra-indication.*—Impermeability of the canaliculus.

### RESULTS.

When I was first asked to undertake treatment for cases of epiphora I adopted the West operation as being the usual procedure for the rhinologist. I performed this on four occasions, all women, two being successful, in one slight epiphora remained, and the fourth was a complete failure, subsequently requiring excision of the sac. Therefore, arguing on my experience of operation on the frontal sinus, I decided that external access would be more likely to prove successful than any intranasal procedure, and I decided to employ the modified Toti operation, together with a submucous resection of the septum where there was marked nasal obstruction. I have performed this operation on eleven cases, ten women and one man, and in nine of these there is now no epiphora, except in high wind, and no discharge from the sac. Of the two failures, in one there was an empyema of the sac which caused infection of the wound and breaking down of the external incision. A second operation failed to establish drainage owing to fibrosis, and excision of the sac was later performed. In the other the opening closed for no obvious reason. In a third case with empyema of the sac a second operation was required for the same reason, but was successful, and the patient has now no trouble.

*Deductions.*—These results in the Toti operation correspond closely with those reported by operators elsewhere over greater numbers of cases. The proportions of cures in the Toti operation is somewhat higher than those generally obtained by the West operation, except in the case of West himself (90 per cent. cures), and of Kofler and Urbanek of Vienna, who obtain better access by operating from the opposite nostril through a window in the septum and claim 100 per cent. cure. I have, however, hesitated to adopt this method, as there is some possibility of subsequent trouble in the nose from a septal perforation.

The conclusions I have drawn from these comparatively few cases are that where there is simple epiphora due to stenosis of the duct, and where there is no active dacryocystitis, the Toti operation is the method of choice, but where there is a closed empyema of the sac which cannot be rendered sterile by previous treatment on the lines indicated by Mr. Iles the West operation should be performed as less likely to lead to infection in surrounding tissues. Excision of the sac should only be employed where these operations have failed, or where there is already considerable matting round the sac.

### BIBLIOGRAPHY.

Meller, J., *Trans. Ophth. Soc. U. Kingdom,* 1929, p. 233.

West, *Archives of Ophthalmology,* 1926, lv., p. 351.

Kofler and Urbanek, *Ztschr. f. Augenh.,* 1925, p. 200.

Henry, L. M. J., *Brit. J. Ophth.,* 1933, xvii., p. 550.

Traquair, H. M., *Trans. Ophth. Soc. U. Kingdom,* 1932, lii., p. 149.

